# Optimism Bias in Firearm-Related Risk Perceptions

**DOI:** 10.1001/jamanetworkopen.2023.49656

**Published:** 2023-12-28

**Authors:** Amanda J. Aubel, Garen J. Wintemute, Nicole Kravitz-Wirtz

**Affiliations:** 1 Violence Prevention Research Program, Department of Emergency Medicine, School of Medicine, University of California Davis, Sacramento

## Abstract

This survey study assesses the perceptions of California adults about the safety and risk of personal firearm use or access vs firearm use or access by others.

## Introduction

Optimism bias, the perception that favorable events are more likely and adverse events less likely to happen to oneself vs similar others, is common across numerous health and safety topics.^1^ In the case of firearms, research suggests that members of households with firearms are, as a group, less likely to worry about sustaining a firearm injury than members of households without guns^2^ despite evidence that firearms in homes elevate firearm injury risk.^3^ However, it remains unclear whether within-individual firearm-related risk perceptions vary depending on whether safety and risk are evaluated for themselves or for others.

## Methods

We conducted the 2018 California Safety and Well-Being Survey^4^ with adult (aged ≥18 years) California residents drawn from the Ipsos KnowledgePanel, an online probability-based research panel including survey-specific weights to produce population-representative estimates. The University of California Davis Institutional Review Board approved this survey study. Participants read informed consent language, and study initiation constituted consent. We followed the AAPOR reporting guideline.

Respondents were classified as gun owners, nonowners in households with guns, and nonowners in households without guns. To assess risk perceptions, we asked respondents, “[does/would] having a gun at your home” and “if everyone in your neighborhood had guns at home” would that make their home or neighborhood, respectively, “safer” or “more dangerous.” Then, we asked, “if you were to carry a gun in public” and “if everyone who legally owned a gun could carry it in public,” would that make their neighborhood “safer” or “more dangerous.” “It depends” and “don't know” were also response options (discussed elsewhere^4^).

We used standard descriptive techniques and weighted repeated-measures multinomial logistic regression to assess within-individual differences in risk perceptions of firearm ownership and carrying for themselves and for others, overall and by firearm ownership status. All statistical tests were 2-tailed, with *P* < .05 indicating significance. Analyses were performed between April 2020 and September 2021, using Stata 15.1 (StataCorp LLC).

## Results

Of 5232 panel members invited to participate, 2558 completed the survey (49% completion rate). Among respondents (1469 females [52.2%], 1089 males [47.8%]; mean [SD] age, 47.8 [17.0] years), 25.1% (95% CI, 22.5%-27.8%) said having a gun at their home made it safer, including 60.6% (95% CI, 53.3%-67.4%) of gun owners, 49.6% (95% CI, 40.0%-59.3%) of nonowners in households with guns, and 14.0% (95% CI, 11.6%-16.9%) of nonowners in households without guns (Table 1). Regardless of gun ownership status, respondents were significantly more likely to say guns made their own home safer than to say guns in others’ homes made their neighborhood safer. Conversely, respondents were significantly more likely to perceive guns in others’ homes as more dangerous than guns in their own home.

**Table 1.** Perceptions of Safety and Risk of Household Gun Ownership for Self and for Others, by Gun Ownership Status (N = 2558)

	No. (weighted %) [95% CI]^a^		Difference	*P* value
	[Does/would] having a gun at your home make it …?	If everyone in your neighborhood had guns at home, would that make your neighborhood …?		
**All respondents**				
Safer	638 (25.1) [22.5-27.8]	442 (16.7) [14.6-19.0]	8.4	<.001
More dangerous	768 (28.6) [25.9-31.4]	1143 (44.0) [40.9-47.1]	−15.4	<.001
It depends	411 (16.8) [14.6-19.3]	383 (16.1) [13.8-18.6]	0.8	.67
Don’t know	730 (28.9) [26.1-31.8]	572 (22.2) [19.7-24.8]	6.7	.001
**Gun owners**				
Safer	261 (60.6) [53.3-67.4]	191 (47.1) [40.0-54.3]	13.5	.009
More dangerous	19 (4.4) [2.4-8.1]	60 (13.4) [9.0-19.4]	−9.0	.003
It depends	78 (21.5) [15.5-28.9]	97 (21.3) [16.0-27.8]	1.5	.97
Don’t know	67 (13.0) [9.4-17.8]	77 (17.2) [12.6-23.1]	−4.2	.22
**Nonowners in households with guns**				
Safer	95 (49.6) [40.0-59.3]	45 (25.8) [17.6-36.1]	23.8	.001
More dangerous	23 (5.3) [2.8-9.8]	74 (23.2) [16.7-31.3]	−18.0	<.001
It depends	54 (15.4) [10.7-21.6]	62 (25.8) [18.0-35.6]	−10.5	.048
Don’t know	70 (29.8) [21.7-39.3]	61 (25.2) [17.9-34.2]	4.6	.45
**Nonowners in households without guns**				
Safer	251 (14.0) [11.6-16.9]	178 (8.6) [6.9-10.7]	5.4	.001
More dangerous	710 (37.7) [34.1-41.3]	991 (54.6) [50.8-58.3]	−16.9	<.001
It depends	273 (16.5) [13.9-19.5]	215 (14.1) [11.6-17.2]	2.4	.23
Don’t know	560 (31.7) [28.2-35.4]	404 (22.1) [19.2-25.3]	9.6	<.001

^a^ Column percentages may not sum to 100% because refusals (<1% of respondents) are not shown.

Compared with their beliefs about firearms at home, respondents less often said that carrying a gun in their neighborhood made it safer; no significant differences were observed between perceptions of safety for themselves vs others (Table 2). Only nonowners in households without guns were significantly more likely to report that gun carrying by others made their neighborhood more dangerous than their own gun carrying.

**Table 2.** Perceptions of Safety and Risk of Gun Carrying for Self and for Others, by Gun Ownership Status (N = 2558)

	No. (weighted %) [95% CI]^a^		Difference	*P* value
	If you were to carry a gun in public in your neighborhood, would that make it …?	If everyone who legally owned a gun could carry it in public in your neighborhood, would that make it …?		
**All respondents**				
Safer	399 (15.6) [13.5-17.9]	383 (15.2) [13.1-17.6]	0.4	.81
More dangerous	1203 (44.6) [41.6-47.7]	1365 (51.2) [48.1-54.3]	−6.6	.003
It depends	169 (7.7) [6.0-9.7]	175 (7.3) [5.8-9.2]	0.4	.78
Don’t know	776 (31.5) [28.6-34.4]	612 (25.0) [22.4-27.8]	6.5	.001
**Gun owners**				
Safer	152 (36.5) [29.9-43.5]	157 (37.2) [30.6-44.4]	−0.8	.88
More dangerous	115 (25.0) [19.4-31.7]	126 (29.3) [22.8-36.8]	−4.3	.37
It depends	44 (10.6) [6.9-16.0]	40 (12.1) [8.1-17.7]	−1.5	.65
Don’t know	115 (27.5) [21.4-34.7]	102 (20.6) [16.2-26.0]	6.9	.10
**Nonowners in households with guns**				
Safer	41 (20.1) [13.1-29.6]	33 (18.2) [11.4-27.7]	2.0	.74
More dangerous	99 (33.7) [25.4-43.0]	108 (38.1) [29.4-47.7]	−4.5	.49
It depends	20 (14.3) [7.8-24.8]	30 (13.8) [8.1-22.5]	0.5	.92
Don’t know	82 (31.9) [24.0-41.0]	70 (29.8) [21.7-39.3]	2.1	.74
**Nonowners in households without guns**				
Safer	188 (10.6) [8.5-13.2]	173 (10.1) [8.0-12.8]	0.5	.77
More dangerous	971 (51.7) [47.9-55.5]	1107 (59.2) [55.4-62.9]	−7.5	.006
It depends	100 (6.3) [4.6-8.7]	100 (5.4) [3.9-7.5]	0.9	.52
Don’t know	534 (31.2) [27.8-34.8]	403 (24.3) [21.1-27.8]	6.9	.005

^a^ Column percentages may not sum to 100% because refusals (<1% of respondents) are not shown.

## Discussion

This general population survey extends prior research^5^ on risk perceptions among gun owners by demonstrating that regardless of gun ownership status, individuals evaluate the benefits conferred and risks imposed by household firearm ownership more favorably for themselves than for others in their neighborhood, signifying optimism bias. Only nonowners in households without guns showed optimism bias in their assessment of gun carrying.

Limitations of this survey study may include social desirability bias and limited generalizability beyond California, which has relatively stringent gun laws, especially regarding gun carrying. Nonetheless, research on other health and safety issues has shown that optimism bias affects whether individuals support and engage in precautionary policies and practices.^1,6^ Thus, to inform prevention strategies, future research should examine whether and how optimism bias affects behaviors associated with firearm injury risk, such as firearm storage.

## Notes

Supplement.
Data Sharing Statement
