# Supplementary material for: Optimism Bias in Firearm-Related Risk Perceptions
Source: JAMA Netw Open. 2023 Dec 28;6(12):e2349656. doi: 10.1001/jamanetworkopen.2023.49656 (PMC10755601; doi:10.1001/jamanetworkopen.2023.49656)
Supplement: Supplement. — Data Sharing Statement [file jamanetwopen-e2349656-s001.pdf]

# Data Sharing Statement

Aubel. Optimism Bias in Firearm-Related Risk Perceptions. *JAMA Netw Open*. Published December 28, 2023. doi:10.1001/jamanetworkopen.2023.49656

**Data**
**Data available:** No
